# Nemaline myopathy with scoliosis: a case report

**DOI:** 10.3389/fped.2024.1413096

**Published:** 2024-10-15

**Authors:** Jin Huang, Chen Zhang, Jing Li, Huaming Wang, Xiaojuan Cui, Juan Wang, Borong Zhang, Xiaoqiang Wang, Deming Lin, Jun Zhao, Jiantao Wen

**Affiliations:** Gansu Provincial Hospital of Traditional Chinese Medicine, Lanzhou, Gansu, China

**Keywords:** nemaline myopathy, scoliosis, muscle weakness, NEB gene, clinical

## Abstract

Nemaline myopathy (NM) is a rare congenital muscle disease that leads to muscle damage, resulting in muscle weakness and atrophy. Cases of scoliosis induced by muscle weakness and atrophy are exceedingly uncommon. The author clinically treated one patient with NM complicated by scoliosis and analyzed its clinical characteristics through a literature review. The pathogenic genes of this patient originated from compound heterozygous mutations c.12471 + 3A>G from the mother and c.7727G>A from the father, leading to the diagnosis of NM accompanied by scoliosis, which represents a relatively rare clinical phenotype.

## 1 Introduction

Nemaline myopathy (NM) is an extremely rare congenital myopathy with genetic heterogeneity (1). This condition is characterized by its extremely low prevalence and the diverse genetic mutations that contribute to its onset and progression. Shy et al. (2) first reported this disease in 1963 and described its clinical features, namely, the presence of rod-shaped structures in muscle fibers. Epidemiological statistics indicate an incidence rate of 1/26,000 (3). The international collaborative group has classified NMs into 6 types, with 13 identified pathogenic genes (4–9), among which mutations in the NEB gene account for 50% of the diagnosed cases (1). The clinical manifestations of NM primarily involve muscle weakness, often affecting muscles such as the neck flexors, limb muscles, respiratory muscles, and facial muscles, with progressive worsening of muscle weakness (10). Complications such as scoliosis may occur as a compensatory response when back muscles cannot function normally (11). With advances in genetic testing technology, invasive diagnostic methods have largely been replaced for diagnosing this disease (12). Here, we present the clinical data of one patient who was diagnosed with NM combined with scoliosis and associated restrictive ventilatory impairment through genetic testing and who was treated at our hospital, along with a review of the relevant literature.

## 2 Materials

A patient (proband) who was admitted to the Juvenile Bone Disease Center of Gansu Provincial Hospital of Traditional Chinese Medicine on January 29, 2023, due to “baculoid myopathy complicated with scoliosis”, was selected as the study object. This study passed the review of the Ethics Committee of our hospital (2023-048-01), and both parents of the proband signed informed consent for the clinical study.

**Figure 1:**
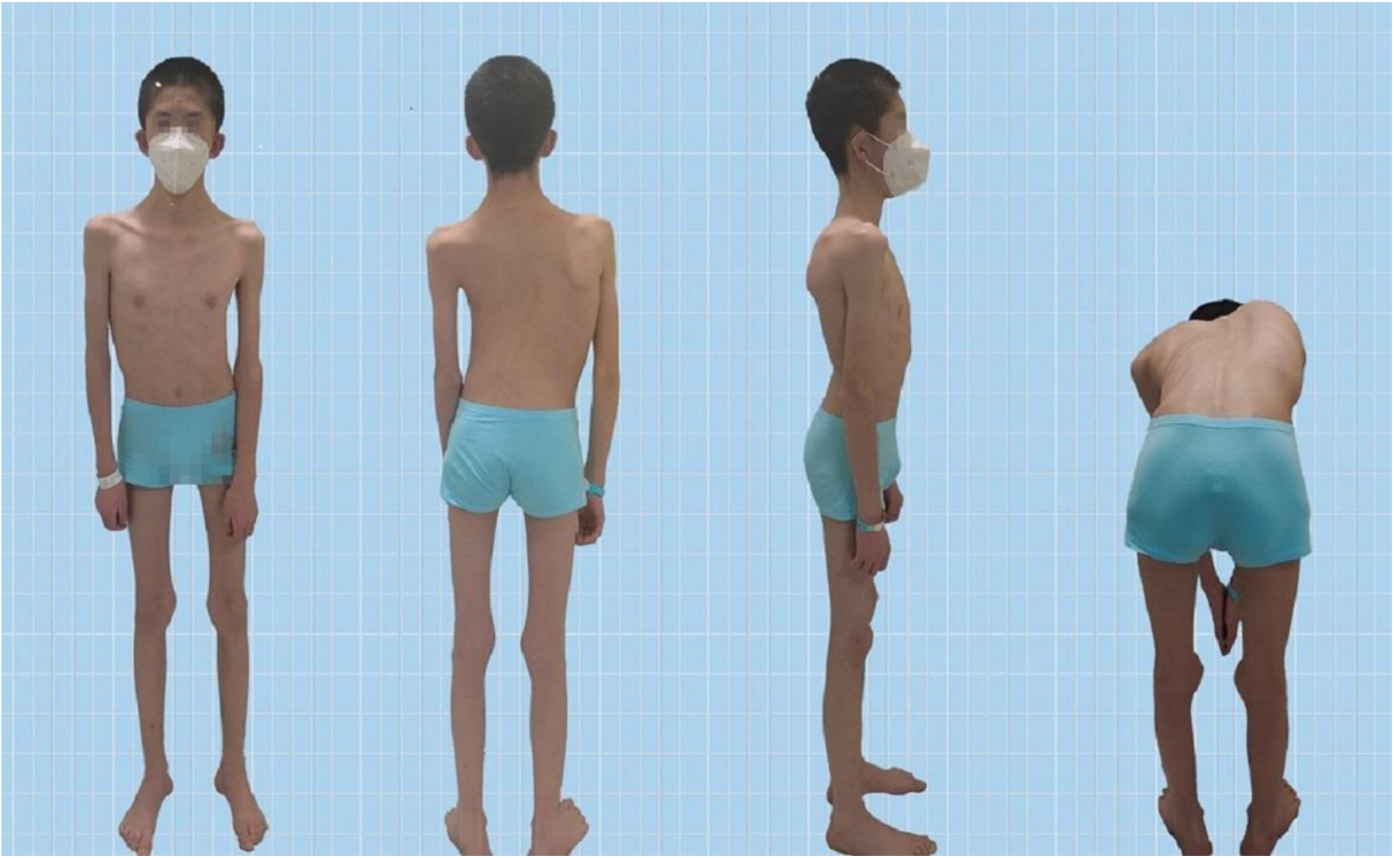
Patient appearance.

The Pediatric Orthopedics Department at Gansu Provincial Hospital admitted a 14-year-old male patient with scoliosis. The patient's parents noticed a bulge on one side of his waist and back two years ago but did not pay much attention to this abnormality. One year prior, they observed the tilting of the patient's body to the left. Recently, this tilting phenomenon has worsened, prompting visits to Gansu Provincial Hospital. Upon admission, the patient presented with a BMI of 13.01, weighing 35.0 kg and measuring 164 cm in height. The patient's skin showed no café-au-lait spots or fibrous tissue (Figure 1). The parents denied any family history of hereditary diseases and reported no consanguinity. The patient was delivered at full term via cesarean section and was fed artificially after birth. Language development begins at the age of 2, which is slightly delayed compared with that of peers. The individual began walking independently at the age of two and had normal intellectual development. However, these patients have weaker muscle strength than their peers do and are unable to sustain prolonged physical activity. When sitting, patients often support their heads with both hands.

## 3 Methods

### 3.1 Motor function evaluation

Muscle strength testing, graded by the Medical Research Council (MRC), revealed limited voluntary movement in the shoulder and neck region, along with weak neck muscle strength. The MRC score for neck flexors was only 2, indicating that the patient habitually supported their head with both upper limbs while sitting. Furthermore, muscle strength was lower in all muscle groups than in age-matched individuals (Table 1).

**Table 1:** Examination of muscle strength in various muscle groups*.*

Muscle group	MRC score	Muscle group	MRC score
Neck flexors	3	Neck extensor muscles	3
Biceps brachii	4	Triceps brachii	3
Extensor carpi radialis muscles	4	Hip extensors	4
Wrist flexor	4	Hamstring muscles	4
Thigh muscles	4	Dorsiflexor muscles	5
Plantarflexor	4	Plantar flexor muscles	4

### 3.2 Non–muscle-related comorbid conditions

#### 3.2.1 Scoliosis

**Figure 2:**
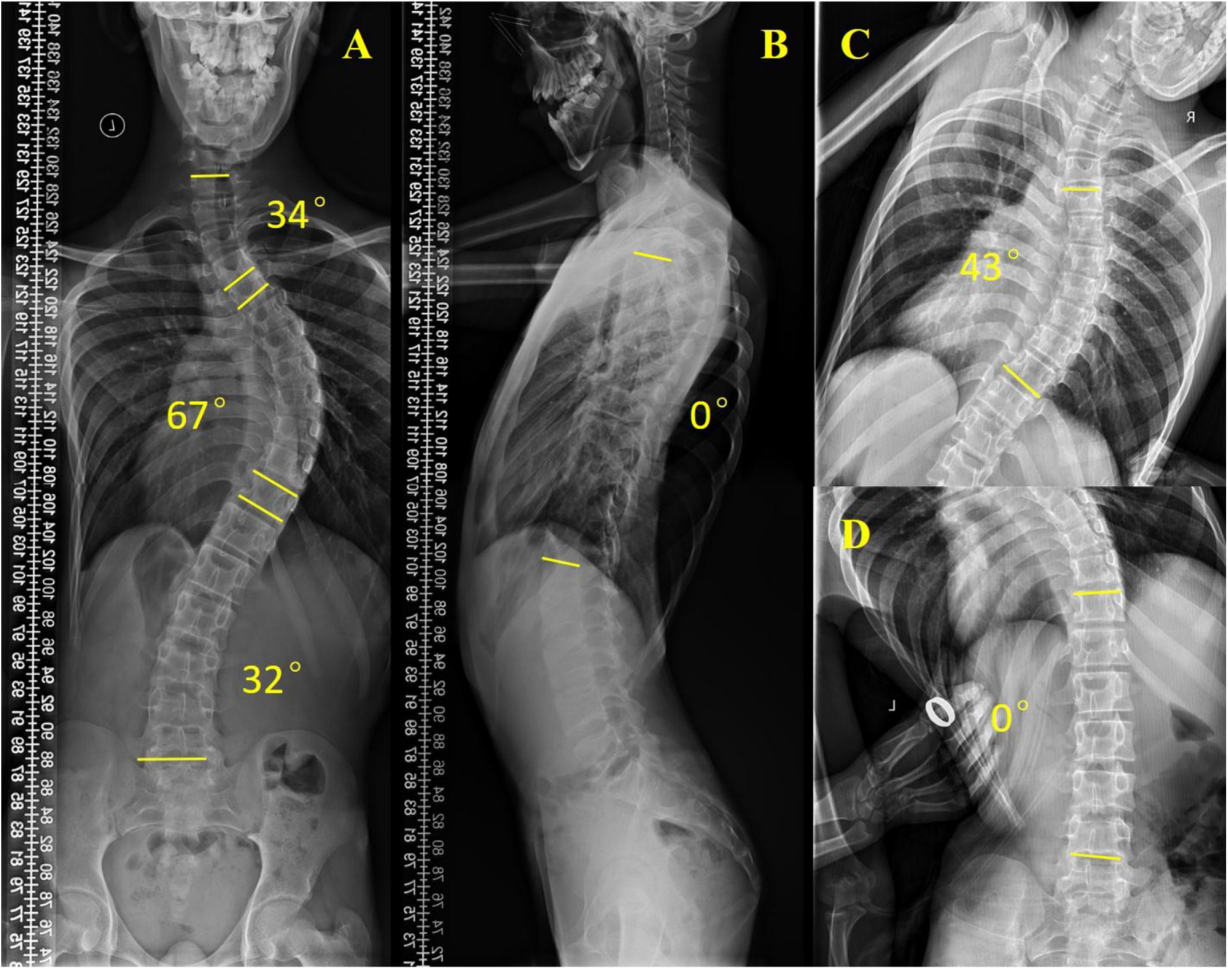
Preoperative x-ray examination. **(A)** Whole-spine anterior film (preoperative), PT: 34°, MT: 67°, TL/L: 32°; **(B)** Full-length lateral spinal radiography, TK: 0°; **(C)** Spine bending x-ray films, MT: 43°; **(D)** Spine bending x-ray films, TL/L: 0°.

**Figure 3:**
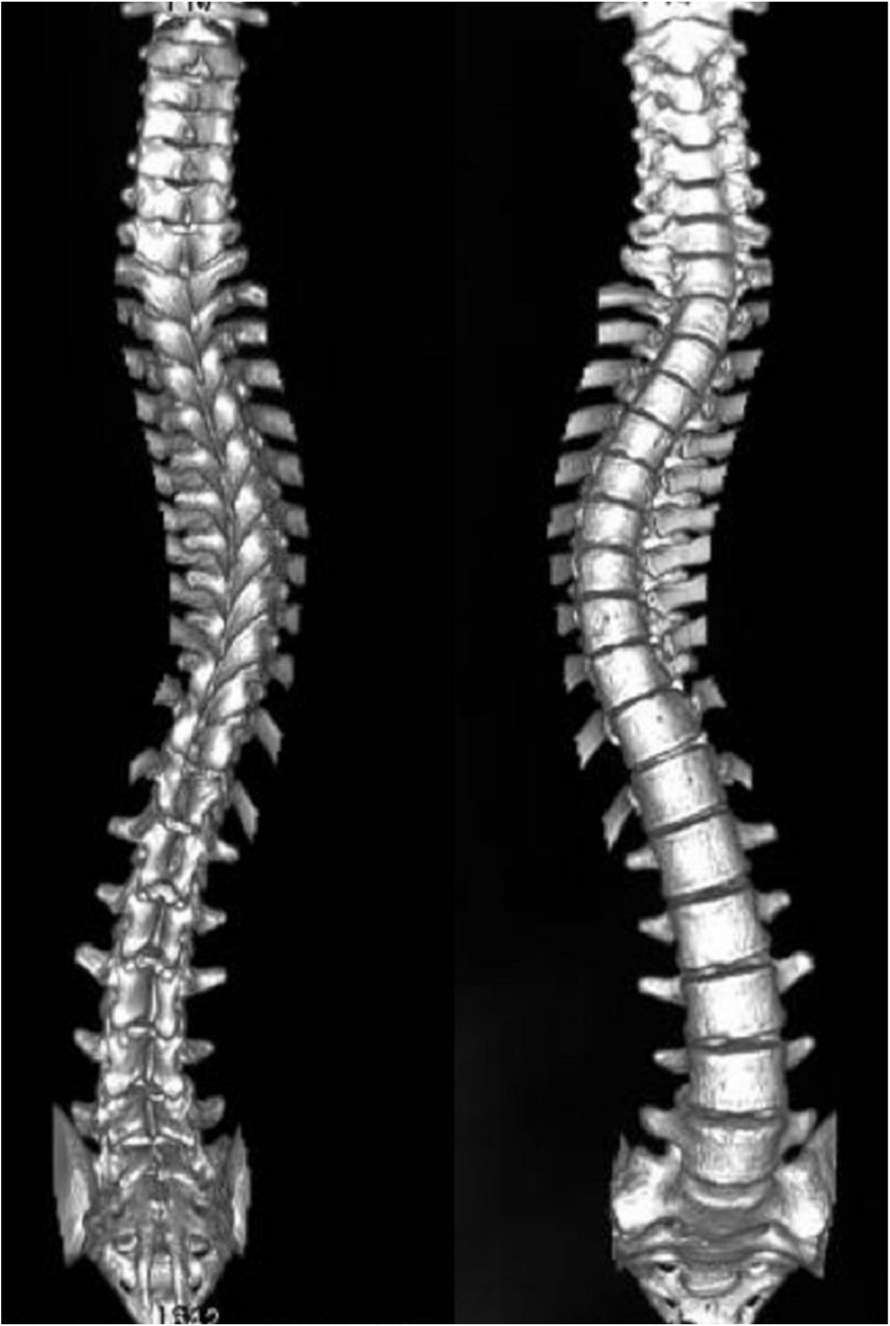
3d reconstruction of the patient's entire spine via CT.

**Figure 4:**
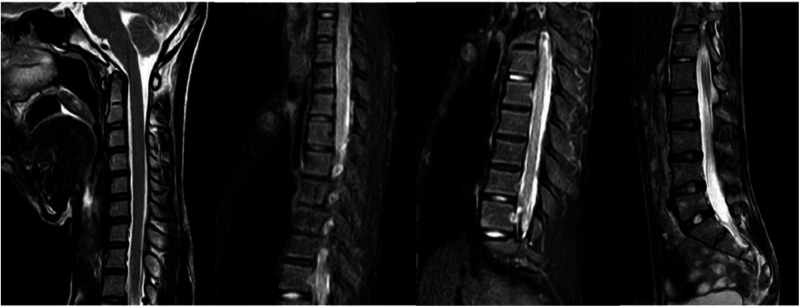
Cervical, thoracic and lumbar spine MRI.

Scoliosis is a common additional phenotype of NM. The patient presented with symmetrical shoulders, with left convexity in the thoracic region and significant right convexity in the lumbar region. No pelvic tilt was observed. The patient also exhibited an evident “razor back” deformity, with bony prominence on the right upper chest, fullness on the right side of the rib cage, and concavity in the right rib waist area. The spine has an “S”-shaped appearance, with no abnormalities in skin sensation, symmetrical physiological reflexes, or elicited pathological reflexes (Figure 1). X-ray revealed scoliosis with a proximal thoracic (PT) angle of 34°, a main thoracic (MT) angle of 67°, and a thoracolumbar/lumbar (TL/L) angle of 32°. Spine bending x-ray revealed a main thoracic spine (MT) angle of 43°, a thoracolumbar/lumbar spine (TL/L) angle of 0°, and a thoracic kyphosis (TK) angle of 0° (Figure 2). A full-spine CT scan revealed no hemivertebra formation or segmental defects in the spine, but some vertebral arches were underdeveloped, ruling out congenital scoliosis (Figure 3). MRI of the patient's full spine revealed no evidence of Chiari malformation, longitudinal splitting of the spinal cord, syringomyelia, tethered cord, or spinal tumors (Figure 4). However, severe scoliosis was observed.

#### 3.2.2 Maxillofacial deformities

The patient presented with a range of craniofacial abnormalities, including facial elongation, macrostomia, a high-arched palate, inability to close the eyes tightly, poor dental occlusion, inability to inflate the cheeks, and air leakage while whistling. Oral examination revealed an Angle Class III malocclusion, a convex facial profile, a high angle, right mandibular deviation, narrow maxillary and mandibular arches, a high palatal vault, tooth crowding, and misalignment of the dental midlines. These findings suggest the presence of severe dental and craniofacial dysmorphisms (Supplementary Figures S1, S2).

#### 3.2.3 Heart function

Echocardiography was conducted on the patient and revealed normal dimensions of the cardiac chambers and interventricular septum. The interventricular septum and left ventricular wall showed continuous integrity with normal thickness and coordinated motion. Mild tricuspid regurgitation was observed during systole, with a regurgitant length of approximately 2.0 cm and a regurgitant area of approximately 2.3 cm^2^. No significant abnormalities were observed in the morphology, structure, or motion of the remaining valves, indicating mild anomalies in valve development.

#### 3.2.4 Pulmonary function measurements

Quantitative measurements of respiratory function were conducted for the patient. Pulmonary function tests (PFTs) could not be completed. The measurements revealed that the total lung capacity (TLC) was 4.24 L, the vital capacity (VC) was 2.12 L, the forced vital capacity (FVC) was 2.09 L, the percentage of forced vital capacity (FVC%) was 55.1%, the first-second forced expiratory volume (FEV1) was 1.56 L, and the percentage of forced expiratory volume in the first second (FEV1%) was 49.7%, all of which were below the predicted values. After inhaling 200 µg of salbutamol aerosol, there was a 0.7% improvement in the FEV1. The absolute increase in FEV1 was less than 200 ml, indicating severe mixed ventilation impairment.

#### 3.2.5 Bulbar function

The language development of patients is delayed, and their language expression is later than that of their peers. The patient also presented with symptoms of drooling. The presence of drooling symptoms was evaluated via the Drooling Severity and Frequency Scale (DSFS), which ranks the severity of drooling on a scale from 1 (dry) to 5 (profuse), with a score of 3 (moderate); the frequency of salivation ranges from 1 (none) to 4 (often), and the measured result is 3 (sometimes). The results of the Kubota drinking water test were at level 2, indicating that the water can be swallowed without choking after being divided two or more times. This suggests a slight impairment in the patient's bulbar function.

#### 3.2.6 Gene mutation testing

Three milliliters of peripheral blood samples were collected from the children and their parents and anticoagulated with EDTA. Genomic DNA was extracted with a kit [Tiangen Biochemical Technology (Beijing) Co., Ltd.] and the DNA concentration and purity were measured with a NanoDrop 2000 UV‒Vis spectrophotometer (Thermo Fisher Scientific, USA), after which the samples were stored at −20°C. Target sequence capture technology was used to construct a library, the target sequence was captured through probes, and high-throughput sequencing was performed on the DNBSEQ-T7 platform (Shenzhen MGI Technology Co., Ltd.). Primers were designed based on the sequencing results, and Sanger sequencing family verification was performed to verify the results. Carry out comparative analysis.

#### 3.2.7 Bioinformatics analysis

After quality control, the raw data were removed from adapters and low-quality short-read sequences and then compared with the reference genome hg19 in the UCSC database via BWA software. The sequencing data were filtered and screened to obtain high-quality variants. Moreover, protein function prediction software was used (PolyPhen-2, PROVEAN), the Human Gene Mutation Database (HGMD), and the Human Mendelian Inheritance Database (Online Mendelian Inheritance in Man, OMIM). The amino acid conservation and protein structure changes of new missense variants were analyzed. Moreover, the pathogenicity of the candidate variants was analyzed according to the relevant guidelines of the American College of Medical Genetics and Genomics (ACMG).

## 4 Results

### 4.1 Genetic test results

Peripheral venous blood samples were collected from the patient and their parents for high-throughput sequencing of the peripheral blood exome, with the informed consent of the patient's family. Two mutations were detected in the NEB gene: (i) a heterozygous mutation, c.7727G>A, at the chr2:152500561 locus and (ii) a heterozygous mutation, c.12471 + 3A>G, at the chr2:152389953 locus. Sanger sequencing validation revealed that the two heterozygous mutations in the child were from his mother and father (Supplementary Figure S3). The corresponding databases include the following disease-related information: bioinformatics analysis software Polyphe-2, MutationTaster, and GERP + predicted that the c.7727G>A (p.W2576*, 5950) and c.12471 + 3A>G heterozygous mutations are likely to be pathogenic. A definitive diagnosis of the NM2 type was made following ACMG guidelines. Additionally, other variants, including c.4796G>A (p.W1599*,449), c.12471 + 3A>G, c.1531A>C (p.K511Q), and c.3709C>T (p.R1237W), were identified, which help explain the child's clinical features, such as delayed speech and language development, as well as delayed motor development (Table 2).

**Table 2:** Clinical phenotype and its relationship with genetic mutation sites.

Deformity	Family history/genetic pattern	Testing tissue	Gene mutation site
High palatal arch, long face shape, low ear position	None/disseminated	Peripheral blood	c.7727G>A (p.W2576*, 5950)
protruding ears	None/disseminated	Peripheral blood	c.4796G>A (p. W1599*,449)
Respiratory disturbance	None/disseminated	Peripheral blood	c.12471 + 3A>G
Scoliosis	None/disseminated	Peripheral blood	c.7727G>A (p.W2576*, 5950)
Muscle weakness	None/disseminated	Peripheral blood	c.7727G>A (p.W2576*, 5950)
Delayed language development	None/disseminated	Peripheral blood	c.1531A>C (p. K511Q)
Delayed motor development	None/disseminated	Peripheral blood	c.3709C>T (p. R1237W)

### 4.2 Treatment plan

According to our analysis of the patient's condition, the main cause of severe mixed ventilatory impairment is the curvature of the spine compressing the lung tissue, leading to a reduced thoracic volume due to a decreased longitudinal diameter of the chest. To address this issue, we proposed surgical correction of spinal deformities in the T3‒L2 region following the principles of adolescent idiopathic scoliosis correction. Postoperatively, the patient's spinal function recovered well, and the degree of body tilt significantly improved (Supplementary Figure S4).

### 4.3 Follow-up

One and a half years after the operation, we followed the patient and measured respiratory function quantitatively: the patient's lung function improved. Among the measurements, the actual measured vital capacity (VC) was 2.62 L, the forced vital capacity (forced vital) was 2.62 L, the capacity (FVC) was 2.6 L, the percentage of forced vital capacity (FVC%) was 56.7%, the first-second forced expiratory volume (FEV1) was 2.38 L, and the percentage of forced expiratory volume in the first second (FEV1%) was 62.8%. Both of these significantly improved from before, indicating a moderately restrictive disorder in pulmonary ventilation function. The child's spinal function recovered well after the operation. After one year of functional exercise, the child's body tilt and other undesirable appearances also greatly improved (Supplementary Figure S5 for details of the status and appearance of the spine during the 1.5-year follow-up period after the operation).

## 5 Discussion

NM is a rare congenital heterogeneous muscle disease. In recent years, genetic testing has become an important method for diagnosing congenital muscle diseases. Currently, mutations in 13 genes, including NEB (13), ACTA1 (14), TPM3 (15), TPM2 (15), RYR1 (16), TNNT1 (17), CFL2 (18), KBTBD13 (19), KLHL40 (20), KLHL41 (21), LMOD3 (22), MYPD (23), and RYR3 (6), have been shown to cause NM (Table 3). NEB gene variation is the most common cause of NM and is typically inherited in a recessive manner (24). This gene consists of a total of 183 exons and encodes nebulin, which is one of the largest proteins found in human sarcomeres and is an essential component of thin myofilaments (25). Nebulin plays a crucial role in the normal assembly and contraction of skeletal muscles (26). Abnormal expression of nebulin can result in hypotonia, muscle weakness, and, in some cases, respiratory failure, leading to death (10, 27).

**Table 3:** Nemaline myopathies, genetic causes and inheritance patterns.

Gene	Protein	Inheritance
NEB	Nebulin	AR
ACTA1	α-skeletal actin	AD, AR
TPM3	Slow α-tropomyosin	AD, AR
TPM2	β-tropomyosin	AD, AR
RYR1	Ryanodine receptor 1	AR
TNNT1	Slow troponin T	AR
CFL2	Muscle specific cofilin	AR
KBTBD13	Muscle-specific ubiquitin ligase	AD
KLHL40	Kelch-like family member 40	AR
KLHL41	Kelch-like family member 41	AR
LMOD3	Leiomodin 3	AR
MYPD	Myopalladin	AR
RYR3	Ryanodine receptor 3	AR

AD, autosomal dominant; AR, autosomal recessive.

Based on the results of high-throughput sequencing, we analyzed the correlation between genotype and phenotype. The mutations most closely associated with pathogenesis are c.7727G>A (p. W2576*, 5950) and c.12471 + 3A>G. The mutation c.7727G>A (p. W2576*, 5950) is of particular interest because of its strong correlation with pathogenesis. The disease associated with c.7727G>A (p. W2576*, 5950) is the NM2 type. The main symptoms of this gene locus include cerebral facies deformities, oral abnormalities, thoracic scoliosis, myopathy-induced respiratory insufficiency, generalized muscle weakness, gait across thresholds, fatigue, and plantar muscle atrophy (28, 29). These clinical manifestations are crucial for all NM patients (30), and the clinical phenotype of the child is highly similar to that of this type of patient. The mutation c.12471 + 3A>G is associated mainly with developmental delay and weakness of the facial muscles, respiratory muscles, neck flexor muscles, and distal limb muscles (31). The clinical manifestations of the patient, including delayed language expression compared with peers, late independent walking, weak muscle strength since childhood compared with peers, inability to sustain prolonged physical activity, and habitual use of both hands to support the head in a seated position, are highly similar to those of this type. Additionally, the patient presented with restrictive ventilatory dysfunction. The patient also exhibited delayed speech and language development as well as delayed motor development. Based on the sequencing results, we consulted the OMIM database and inferred that these two phenotypes might be associated with the mutation c.1531A>C (p.K511Q) (32).

Currently, there is no known cure for NM. The goal of treatment is to alleviate symptoms, prevent complications, and provide multidisciplinary interventions to reduce patient pain. Some studies (33) have suggested that L-tyrosine may alleviate symptoms, but large-scale clinical trials have not yet been conducted. The results of genetic detection of NMs may provide potential therapeutic methods. In this case, the patient had a Cobb angle of 67°, a grade 4 iliac Risser sign, and respiratory dysfunction. Surgery and orthopedics are needed to restore limb balance, expand thoracic volume, and improve respiratory function, which significantly enhances patients’ quality of life.

In summary, the primary clinical symptoms of NM include muscle weakness, particularly in the cervical flexors, proximal limbs, respiratory muscles, and facial muscles. Additionally, scoliosis is a common nonmuscular symptom. The child in this case was diagnosed with NM due to heterozygous mutations in the NEB gene, C.7727G>A and c.12471 + 3A>G, which were inherited from both parents. Therefore, it is important to be vigilant about the possibility of early-onset NM in children with no special birth history, an abnormal facial appearance, or significantly delayed motor development. Genetic testing is the gold standard for diagnosing NM, and it should be performed as early as possible to avoid misdiagnosis and mistreatment.

## Data Availability

The original contributions presented in this study are included in the article/Supplementary Material, and further inquiries can be directed to the corresponding authors.
