# Supplementary material for: Nemaline myopathy with scoliosis: a case report
Source: Front Pediatr. 2024 Oct 15;12:1413096. doi: 10.3389/fped.2024.1413096 (PMC11518715; doi:10.3389/fped.2024.1413096)
Supplement: Supplementary file 1 [file Image1.pdf]

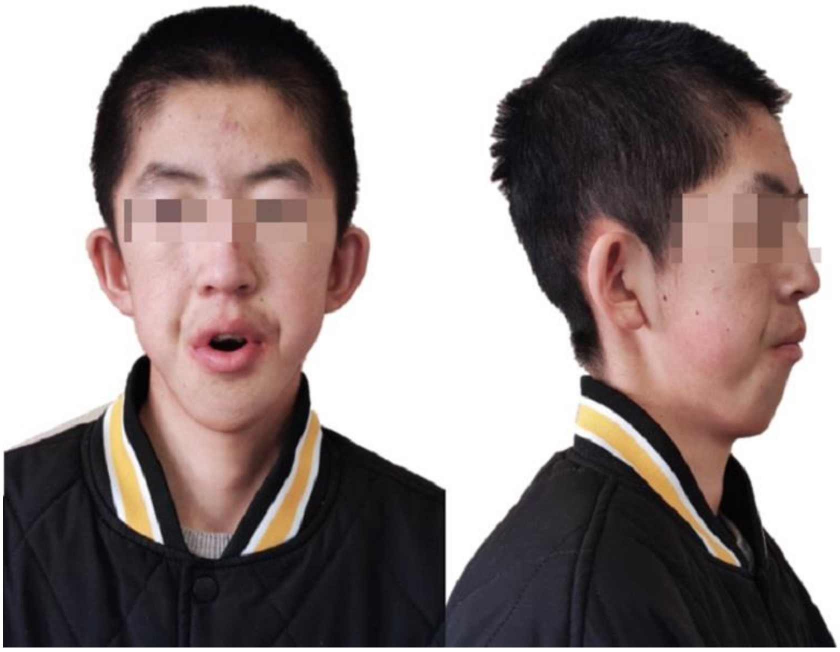

Figure S1. Facial characteristics: elongated facial structure, slender face shape, moderate length
