# Supplementary material for: Nemaline myopathy with scoliosis: a case report
Source: Front Pediatr. 2024 Oct 15;12:1413096. doi: 10.3389/fped.2024.1413096 (PMC11518715; doi:10.3389/fped.2024.1413096)
Supplement: Supplementary file 2 [file Image2.pdf]

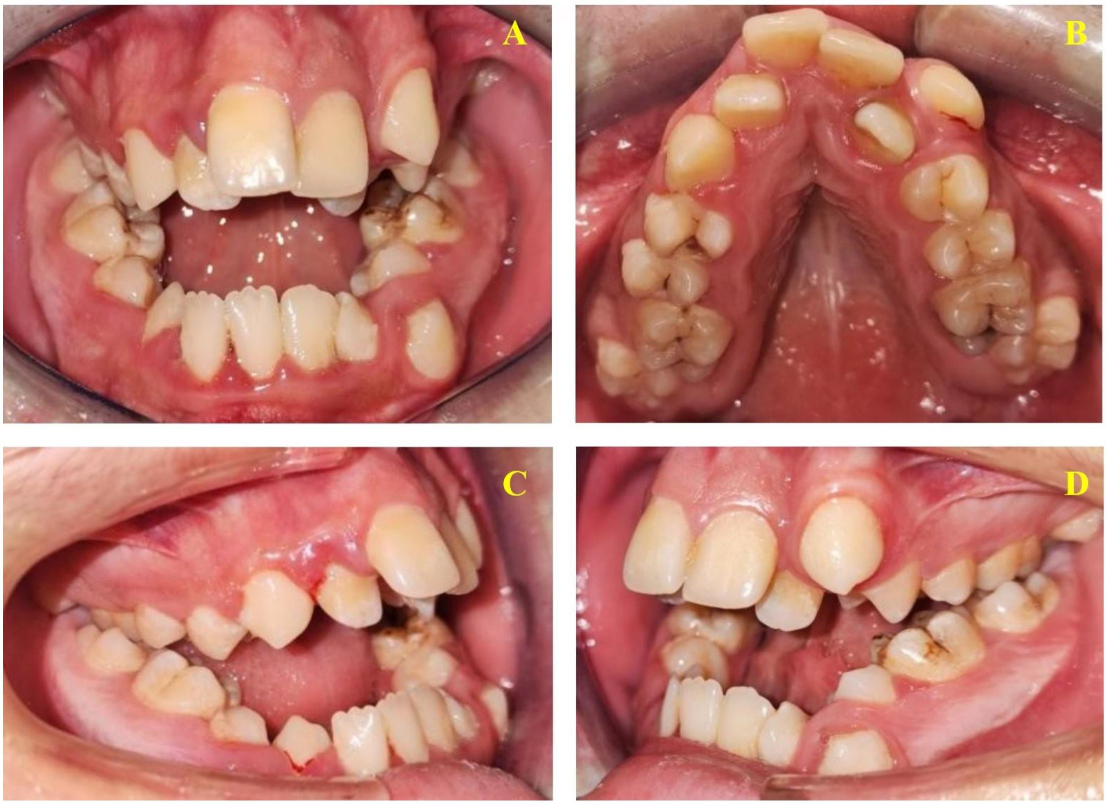

Figure S2. Dental and maxillofacial deformities (A. Angle class III malocclusion with a protrusive mandible, high angle, and right-sided chin deviation; (B). Narrow maxillary and mandibular arches, high palatal vault, crowded dentition, and irregular tooth alignment; (C, D). Posterior crossbites of 16/47 and 26/37, with the remaining dental arches open, and misalignments of the maxillary and mandibular midlines.
