# Supplementary material for: Nemaline myopathy with scoliosis: a case report
Source: Front Pediatr. 2024 Oct 15;12:1413096. doi: 10.3389/fped.2024.1413096 (PMC11518715; doi:10.3389/fped.2024.1413096)
Supplement: Supplementary file 3 [file Image3.pdf]

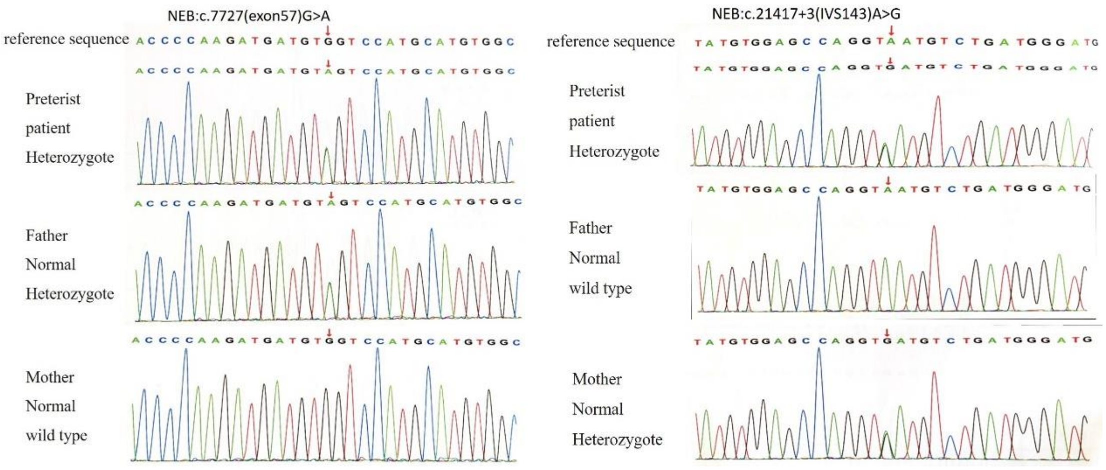

Figure S3. The heterozygous mutations in the patient were inherited from both the mother and the father.
