# Supplementary figures and images for: Nemaline myopathy with scoliosis: a case report
Source: Front Pediatr. 2024 Oct 15;12:1413096. doi: 10.3389/fped.2024.1413096 (PMC11518715; doi:10.3389/fped.2024.1413096)

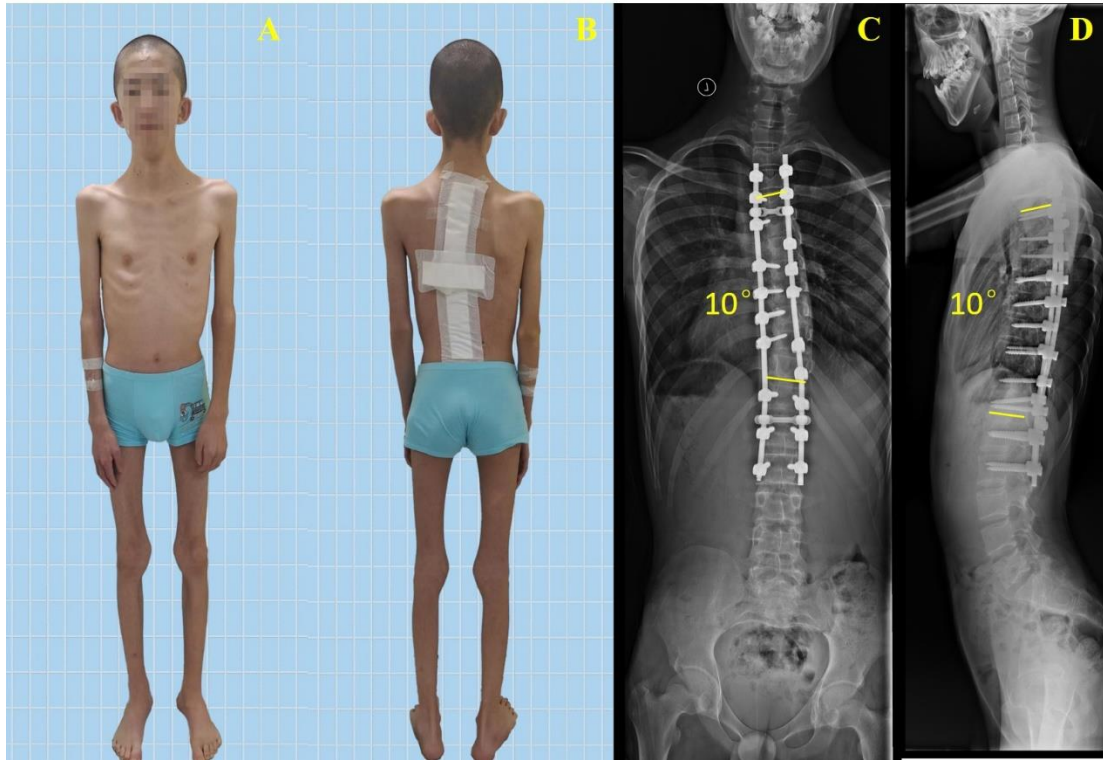

**Figure S4.** Postoperative appearance and X-ray examination revealed the following: MT: 10 °, TK: 10 °.

Supplement: Supplementary file 4 [file Image4.pdf]
