# Supplementary material for: Nemaline myopathy with scoliosis: a case report
Source: Front Pediatr. 2024 Oct 15;12:1413096. doi: 10.3389/fped.2024.1413096 (PMC11518715; doi:10.3389/fped.2024.1413096)
Supplement: Supplementary file 5 [file Image5.pdf]

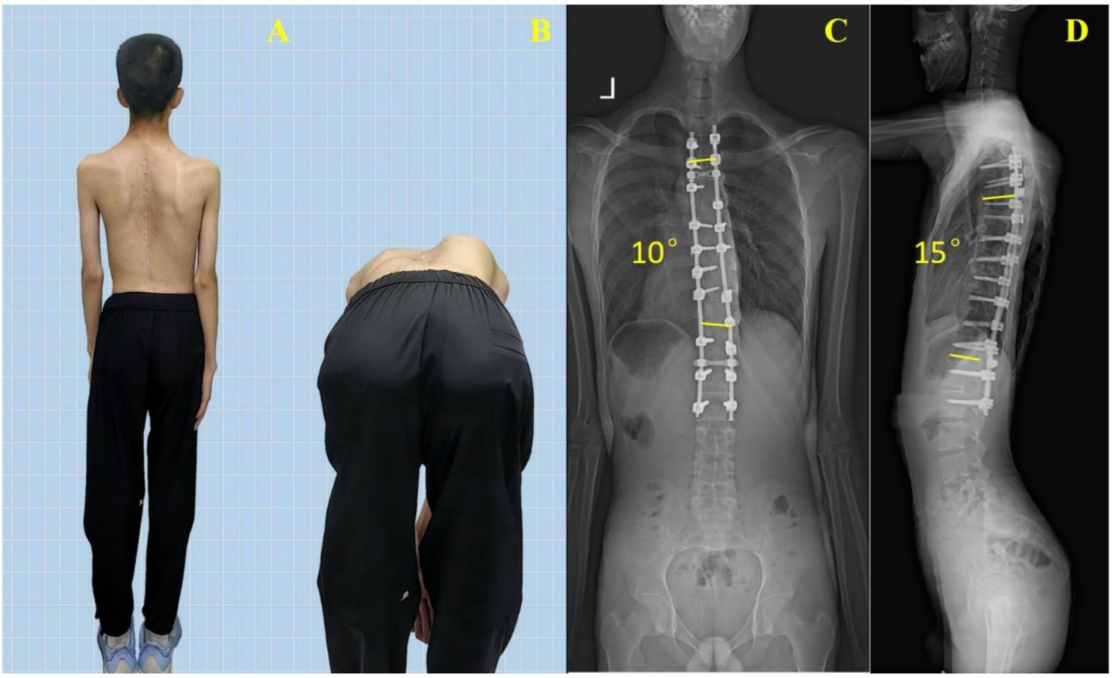

Figure S5. Postoperative follow-up appearance and X-ray examination 1.5 years later revealed that the trunk tilt and razor back deformity significantly improved compared with the preoperative values (MT: 10 °; TK: 15 °).
